# Abundance and accessibility of forage for reindeer in forests of Northern Sweden: Impacts of landscape and winter climate regime

**DOI:** 10.1002/ece3.8820

**Published:** 2022-04-14

**Authors:** Ilona Kater, Robert Baxter

**Affiliations:** ^1^ 3057 Department of Biosciences Durham University Durham UK

**Keywords:** competition, forestry, grazing, ice, reindeer, snow

## Abstract

The survival of reindeer during winter, their period of greatest food stress, depends largely on the abundance and accessibility of forage in their pastures. In Northern Sweden, realized availability of forage is notably affected by snow conditions and the impacts of forestry. While these factors have been examined in isolation, their combined effect has, to the best of our knowledge to date, not been researched. In this study, vegetation surveys and analysis of snow conditions were undertaken in forest stands at various stages of recovery from clear‐cutting. The variation in abundance and growth of understory species edible by reindeer, such as lichen, was noted as forests matured. The barrier effect of ice lenses in the snow was also measured in these stands. Lichen biomass was significantly affected by a combination of stand maturity, understory vegetation height, and lichen height. Soil disturbance from the processes of felling and competition in the vegetation communities recovering from this disturbance were identified as key drivers of change in lichen biomass. Overall, clear‐cut forests had some of the greatest prevalence of ice lenses in the snow column, and forage availability at these sites was up to 61% less than in mature stands over 58 years in age. It is suggested that alternative silviculture methods are investigated for use in this reindeer herding region, as frequent clear‐cutting and consequent reduction in the average forest stand age and maturity class may be detrimental to reindeer grazing, reducing both abundance of forage, and access to it during winter.

## Graphical abstract

This study examines how realized forage for reindeer differs in forests at various stages of recovery from clear‐cutting in the animals winter pastures in Northern Sweden. Realized forage is assessed by studying both forage presence and its accessibility for reindeer through snow.

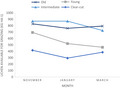

## 1 INTRODUCTION

The availability of a resource to an organism depends largely upon both the abundance of the resource and its accessibility. When assessing the quality of a habitat in relation to a species, both these factors must be considered, as well as how they change through time, especially in harsh regions where small shifts in the environment may result in large impacts on the health and survival of an organism. This is particularly relevant for reindeer (*Rangifer tarandus* L.), whose populations in Northern Europe are contending with large‐scale landscape disturbance from anthropogenic activities, alongside difficult climatic conditions in their ecosystem during winter due to climatic change (Anttonen et al., 2011; Hansen et al., 2011). Semi‐domesticated reindeer populations in this region are not currently experiencing a notable decline in numbers yet there is evidence that their grazing systems are under stress. In Sweden alone, pastures rich in lichen, an important forage for reindeer, have declined by 70% between 1953 and 2013 (Sandström et al., 2016), and since 2002 areas regarded as abundant in lichen have declined by 1% per year (Horstkotte & Moen, 2019). Herders are also having to provide supplementary feed more regularly as their reindeer cannot access sufficient food (Axelsson‐Linkowski et al., 2020; Persson, 2018), creating a growing economic stress which has thrown into question the viability of herding in the long term and has highlighted the need to better understand how and why forage is being lost from the landscape.

### 1.1 Food stress and climate

Reindeer have been present in Northern Fennoscandia since the retreat of the last Fennoscandian ice sheet *ca*. 10,000 years B.P. and have been semi‐domesticated by Indigenous Sámi herders for at least the past millennium (Salmi et al., 2021). In mountain reindeer herding groups in Sweden, reindeer generally migrate west into the Swedish highlands during summer and return eastwards to the lowland boreal forests for the winter. Winter is a notable period of food stress, partly as conditions are not conducive to the growth of plants, typically leading to a lower abundance of forage. Lichens, especially of the genus *Cladonia*, are an important food source for reindeer during this period and can constitute up to 80% of their diet in some areas (Heggberget et al., 2002). Snow also contributes toward this seasonal food stress. Reindeer are well adapted to their environment, being able to detect lichen through up to 91 cm in soft snow (Helle, 1984). However, moving and digging through deep snow has a high energetic cost, which may not be matched by the energy gained from forage lying below. Additionally, if snow hardens into ice, reindeer may be unable to break through and access the underlying food (Axelsson‐Linkowski et al., 2020; Lie et al., 2008). These ice layers form to some extent each winter, often in exposed areas where wind compacts the snowpack over time. However, recent years have seen an increased occurrence of warm periods during winter, with temperatures rising above 0°C, causing precipitation to fall as rain rather than snow. This subsequently freezes into ice when temperatures fall, in what is termed a “rain‐on‐snow” event. Alternatively, surface layers of snow may melt in the warmth and later refreeze to ice, termed as “freeze‐thaw” event (Axelsson‐Linkowski et al., 2020; Hansen et al., 2011). Both freeze–thaw and rain‐on‐snow events cause a far larger and more uniform ice crust to form compared to the patchy ice created by windpack, making forage inaccessible over a wider area. This formation of ice has led to the starvation of hundreds or even thousands of ungulates across the Arctic (Department of Economics, 2015; Hansen et al., 2011; Joly et al., 2009; Lie et al., 2008; Rennert et al., 2009), and icing events are predicted to increase in frequency with a changing climate (Fortin, 2010), so the potentially highly detrimental effects of climate should not be underestimated.

Climatic conditions are not the only factors affecting the ability of reindeer to access food. Anthropogenic development across Fennoscandia has caused widespread loss of reindeer pasture, both through physical presence of infrastructure and reindeer avoidance behavior toward structures such as mines, windfarms, and tourist resorts, all of which have been widely noted in the literature (e.g., Anttonen et al., 2011; Eftestøl et al., 2019; Helle et al., 2012; Hermann et al., 2014; Panzacchi et al., 2013; Skarin et al., 2018). Forestry, a key industry in many reindeer herding areas in Northern Sweden, is another form of land us with notable effects on reindeer grazing.

### 1.2 Forestry

The migration of reindeer in Sweden to lowland boreal forests during the critical winter period makes the state of these forests highly influential to reindeer survival (Käyhkö & Horstkotte, 2017). The counties of Norrbotten, Västerbotten, and Jämtland, where herding primarily takes places (Jacobsson et al., 2020), contain 9.3 million ha of productive forestry lands (SLU, 2018). In some areas, commercial silviculture has caused up to a 50% loss in winter pasture (Berg et al., 2008; Kivinen et al., 2012; Korosuo et al., 2014). As reindeer herding is a traditional livelihood with deep cultural connections for the Indigenous Sámi, reindeer herders have special customary usage rights of public and private land, allowing their reindeer to roam and graze in commercial forests in Sweden (Rennäringslag, 1971). However, individuals and companies who own these forested grazing grounds also have the right to undertake commercial silviculture within these areas, which can alter the landscape in ways that leave little or no forage remains for the reindeer. Therefore, while the reindeer still have the right to be present, these forested areas may be functionally lost to them (Bostedt et al., 2003; Widmark et al., 2013).

Loss of forage can occur in a number of ways: Clear‐cutting, where almost all trees are felled at a site, utilizes heavy machinery such as harvesters and forwarders to fell, delimb, and remove the logs, a disturbance from which the ground vegetation community takes time to recover. Further, during replanting, scarification is often undertaken, where soil is tilled, or turned, to improve tree seedling growth (Saursaunet et al., 2018).

Aside from initial disturbance, commercial forestry also has some longer term effects upon reindeer grazing. As widespread areas are felled and replaced with young plantations, there is a reduction in old growth forests (Horstkotte et al., 2011; Kivinen et al., 2012). These forests are widely recognized as being optimum for grazing as they contain a higher abundance and diversity of lichens compared to younger stands (Horstkotte et al., 2011; Kivinen et al., 2012; Lie et al., 2009; Sandström et al., 2016). This is partially due to the presence of arboreal lichen, found in forests >63 year of age (Horstkotte et al., 2011), which can be accessed without the energetically demanding task of digging through snow. During periods of severe icing, these arboreal lichens can form a vital food source that can help a population to survive (Pekkarinen, 2018).

Much is already understood about the immediate effects of logging, as well as the benefits of mature forest stands. However, a large proportion of Sweden's forests are now at an intermediate stage, where trees have grown back after clearcutting, but have not yet attained the structure of old‐growth forests. Research on these forests of intermediate maturity has established the broad characteristics of lichen biomass and structure (Akujärvi et al., 2014; Uboni et al., 2019), noting how the higher density of trees in younger stands reduces the ability of reindeer to maneuver and dig for lichens. Younger stands are also significantly related to low lichen cover, perhaps partially due to reduced infiltration of light through the dense canopy, affecting growth (Huusko, 2008, Sandström et al., 2016). Nevertheless, the processes of change and recovery of forage species during these intermediate stages of regrowth, especially in winter grazing areas, remain largely unstudied. Even less research exists on the barrier effects of snow for reindeer grazing (e.g., Horstkotte & Roturier, 2013), although there is a growing body of work involving interviews with reindeer herders themselves to document experiential knowledge on this topic (Eira et al., 2018; Reinert et al., 2009; Roturier, 2011; Roturier & Roué, 2009). While valuable in their own right, many of these data are qualitative in nature, and there remains much need for quantification of the key factors and processes.

### 1.3 Aim

The aim of this study was to examine variation in winter forage availability for reindeer according to forest maturity class, based on commercially logged forests in Northern Sweden. Much of this research is observational, noting trends and patterns in the ecology of these forests of differing maturity. However, we also present two hypotheses as follows:

1. Forests which have experienced more recent disturbance from clear‐cutting have a lower abundance of lichen.
2. Forests with greater canopy cover, which can catch snow, contain shallower ground‐lying snow and fewer ice layers in the snow column which are impenetrable to reindeer.

## 2 MATERIALS AND METHODS

### 2.1 Study area

Data were collected near Jokkmokk in Norbotten, Sweden (66°20′N), within an area of approx. 150 km^2^. This area forms part of the winter grazing grounds of three mountain Sámi siidas (Tuorpon, Sirges, and Jåkkåkaskatjiellde), and one smaller forest Sámi siida (Slakka). The topography is undulating hills at *ca*. 250 m above sea level, and the Lilla Luleälven river runs through the area. The landscape is dominated by Scots pine (*Pinus sylverstris* L.) forests, interspersed with Norwegian spruce (*Picea abies* (L.) Karst) and silver birch (*Betula pendula* Roth), with an understory characterized by shrub species such as *Empetrum nigrum* L., *Calluna vulgaris* (L.) Hull and *Vaccinium spp*. over a bed of *Pleurozium schreberi* (Brid.) Mitt and *Dicranum scoparium* Hedw. Lichens are prevalent in the area, mostly consisting of *Cladonia arbuscul*a (Wallr.) Flot., and *C*. *rangiferina* (L.) Weber ex F.H. Wigg., although *C*. *pyxidate* (L.) Hoffm., *C*. *stellaris* (Opiz) Pouzar & Vezda, *Stereocaulon* spp. and *Nephroma arcticum* (L.) Torss. are also present. Arboreal lichen species *Hypogymnia physodes* (L.) Nyl. and *Bryoria fuscescens (*Gyeln.) Brodo & D. Hawksw are also present.

Commercial forestry is widespread in our study area. Forestry inventories from 144 stands within 70 km of our sites show that 33% of the forest stands, including pine, spruce, and mixed stands, are >120 years (Berg, 2008). The remaining 67% are younger, having experienced logging within the last *ca*. century. Sweden's largest forestry company Sveaskog Förvaltnings AB, who have part of their operations in Norrbotten County, routinely using soil scarification to prepare clear‐cut areas for replanting (Bureau Veritas Certification, 2020).

Mean temperatures range from −14.6 °C in January to +14.5 °C in July (Norwegian Meteorological Institute, 2019), though can vary widely, such as from −35 °C in February to +28 °C in June 2019. Mean annual precipitation is 475 mm (Climatemps, 2017).

*Maturity classes based on tree height* For the purpose of this study, 16 forest stands were sampled using a stratified random approach, with four stands being identified for each of the following maturity classes: “clear‐cut” (≥90% of trees felled); “young” (trees 0.5 m–2 m in height, arboreal lichens absent); “intermediate” (trees >2 m in height, arboreal lichens absent); and “old” (trees >2 m in height, arboreal lichens present). To minimize potential edge effects, sampled stands were >20 m from a road or dwelling. The proportion of each maturity class in the nearby area are “clear‐cut” 10% landscape coverage, “young” 35%, “medium/intermediate” 35% and “old” 20%, as seen in equivalent classes measured in Akkajaur/Abraur and Eggelats, Sweden (Berg et al., 2008).

Tree age in each maturity class was assessed using dendrochronological procedures from Grissino‐Mayer (2003). A Mattson No. 4. Increment Borer (Sorbus International, Somerset, UK) was used to extract cores at breast height, or at 50 cm if trees were below breast height, from four randomly chosen trees. Annual growth rings were counted by eye for each tree and an average of the four ages used.

Canopy cover was measured using a spherical densiometer (Spherical Crown Densiometer, Convex Model A, Forestry Suppliers, USA.) according to methods in Werner (2019). Five replicate readings were taken per site, and measurements were made in all four of the “young” sites, plus three each of the “old” and “intermediate” sites as four could not be obtained due to access issues. Clear‐cut areas were not measured due to the absence of a canopy.

### 2.2 Understory vegetation community composition

Vegetation surveys were undertaken during June and July 2019. At each of the 16 forest sites, 10 replicates of 4 m^2^ quadrats (determined as optimum sampling size from a preliminary species‐area curve) were randomly placed >10 m apart. Percentage cover of each lichen and plant species (vascular and bryophytes), bare ground and plant litter was estimated by the lead author for consistency of estimation. Plant height was measured at five random points within each quadrat and averaged. Measurements were taken from the soil surface, or if on moss beds from the point at which the moss started to turn brown (i.e., the point below which photosynthetic material was not present). Lichens on wind‐thrown branches within a quadrat were ignored at this stage.

The plant species present were classified as either “edible” or “non‐edible,” based upon whether reindeer commonly eat them (according to Danell et al., 1994; Inga, 2007; Klein, 1990; Nieminen & Heiskari, 1989). The edible species were *Vaccinium vitis*‐*idaea* L., *V*.*myrtillus* L., *Pleurozium schreberi*, *Cladonia rangiferina* (L.) Weber ex F.H. Wigg., *C*. *arbuscula* (Wallr.) Flot., *C*. *stellaris* (Opiz) Pouzar & Vezda, *C*. *pyxidata* (L.) Hoffm., *Empetrum nigrum* L., *Deschampsia flexuosa* (L.) Trin., *Nephroma arcticum* (L.) Torss. and *Stereocaulon* spp., and the non‐edible were *Dicranum scoparium* Hedwig, *Rhododendron tomentosum* Harmaja, *Vaccinium uliginosum* L., *Polytrichum juniperinum* Hedwig and *Diphasiastrum complanatum* (L.) Holub. It should be noted that there is a variability of preference in edible species, for example, reindeer are not strongly inclined toward eating *Vaccinium myrtillus*, *Pleurozium schreberi* and *Empetrum nigrum*, but will do so when little else is present (Danell et al., 1994).

### 2.3 Lichen surveys

Within two of the 4 m^2^ quadrats at each site described above, five smaller quadrants of 10 × 10 cm (area = 0.01 m^2^) were placed in a stratified random manner on patches dominated by lichens. Lichen cover was noted, along with mean lichen height measured at three random points. All lichen within the 0.01 m^2^ quadrats was extracted, separated from surrounding soil and vegetation, and dried to a constant weight at 105 °C, after which it was immediately cooled and weighed (Satorius M‐prove, Satorius AG, Goettingen, Germany) as in Kumpula et al. (2000). Mean lichen mass across all quadrats within a site was then calculated as kg ha^−1^. Quadrats used here (0.01 m^2^) were smaller than those of 0.25 m^2^ used by Kumpula et al. (2000) to allow for a greater number of replicates within the forest.

Arboreal lichens were only measured at the four “old” sites, being absent by our definition of forest maturity classes in other sites. In each “old” site five trees were chosen at random and their arboreal lichens collected up to the height of 3 m, this being a combination of reindeer browsing height (<1.5 m according to Kumpula et al., 2011) and a maximum local snow depth of 1.2 m (SMHI, 2021b) with comments from locals that some snow patches may reach approx. 1.5 m in depth. Arboreal lichen that had fallen onto the ground, termed “branch lichen” as sometimes still attached to branches, were collected within a 4 m^2^ quadrat adjacent to each of the five trees per site. Arboreal and branch lichens were dried and weighed in the same manner as the terrestrial lichens.

### 2.4 Snow depth, ice layers and presence of cratering

**FIGURE 1:**
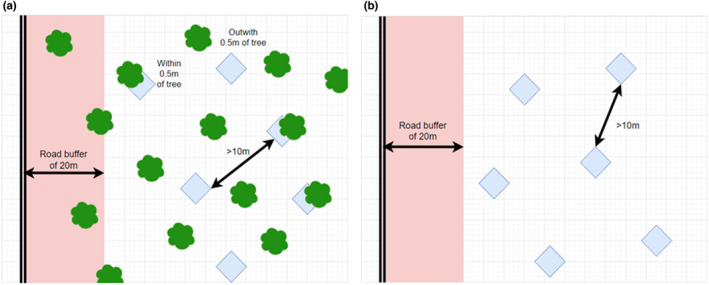
(a) Sampling approach for forested sites. Squares indicate 4 m^2^ quadrats, placed within sites which are >20 m from the road indicated by the double parallel lines, and >10 m from one another. Three quadrats were placed within 0.5 m of a tree, indicated by the curved shapes, and three are placed >0.5 m from a tree in an otherwise random placement. (b) shows sampling approach as applied in clear‐cut areas, where, due to the absence of trees all six quadrants were randomly placed within the site regardless of vegetation

Snow pits were dug in November 2019, plus January and March 2020. At each of the 16 sites, six snow pits were dug >10 m distance from one another. They were placed using stratified random sampling, with three within 0.5 m of a tree and three in open areas between the trees, to adequately capture the effects that the tree canopy exerts upon snow characteristics (Fassnacht et al., 2006). At clear‐cut sites, the six snow pits were randomly distributed due to absence of trees (Figure 1).

Pits were dug to ground level and their depth measured. Hardness of each layer within the snow column was determined using the hand hardness test as described in Höller and Fromm (2010). All measurements were undertaken by the lead author to ensure that, despite the subjective nature of this method, data were collected in a consistent way and the results were directly comparable. The thickness of each distinct layer was measured with a ruler and their position in the snow column noted.

Before digging the snow pits, each site was visually assessed by the author. If greater than 1% of the site showed signs of reindeer cratering, this being the act of digging to vegetation below, the site was classified as having cratering present, otherwise classified as cratering being absent.

### 2.5 Impenetrable ice layers (PKI and båddne ice)

There is no clear published limit of snow hardness through which reindeer can dig. Fancy and White (1985) observed that they can crater through a snow density of at least 2,000 g cm^−2^, although this is highly energetically demanding, whilst an equation created by Skogland (1978) shows that reindeer cease to dig at a snow ram hardness of 1,985 g cm^−2^. These two studies indicate that snow hardness of *ca*. 2,000 g cm^−2^ is on the cusp of the reindeer's ability to crater for food.

For comparability, hand hardness values can be converted to g cm^−2^ by first converting to units of kPa according to Höller and Fromm (2010), and then multiplying these figures by 10.197. Comparing the 2,000 g cm^−2^ cusp of reindeer cratering ability with hand hardness values broadly indicates that reindeer grazing is severely impacted (either highly energetically costly or impossible), in the snow hardness categories “pencil,” “knife,” and “ice”—from here on collectively termed PKI layers. Whilst the ability of reindeer to break through ice depends on multiple factors, for the purposes of this study it was deemed that if these PKI layers are in the lowest level of the snow column, they are impenetrable to reindeer. This is based off TEK of Sámi reindeer herders who identify this layer of snow, termed *båddne*, as specifically difficult to break through when frozen. Therefore, here any sites with *båddne* PKI layers were deemed to be totally inaccessible to reindeer for grazing.

### 2.6 Interannual variation in snow conditions

Snow data were unavoidably limited to measurements made during the winter of 2019/20 (Covid‐19 limitations on movement prevented sampling during winter 2020/21). To assess representativeness of the conditions during the winter of 2019/20, analyses of meteorological data were undertaken. Daily temperature data at Jokkmokk flygplats weather station was available for winters starting 2006 until the winter of 2019/20, accessed from the Swedish Meteorological and Hydrological Institute (SMHI, 2021a), alongside daily snow depth data during the same period at Murjek D weather station (SMHI, 2021b). Jokkmokk flygplats sits approx. 15 km southeast from Jokkmokk town, and all field sites are between these two locations, whilst Murjek D weather station is located 50 km east of Jokkmokk and 32 km east of Jokkmokk flygplats.

Winter was defined as 1st October to 20th April corresponding to the period when reindeer are allowed to be present in their winter grazing grounds (Rennäringslag, 1971). For each winter the maximum, minimum and mean temperatures were noted. The number of days >0°C as well as the number of passages of 0°C that occurred during each winter was counted. The maximum and mean snow depth was also noted, along with the dates and number of days of consistent snow cover (>3 consecutive days of >0 cm snow). Finally, the number of winter warming events for each year was calculated, these being regarded as a decrease in snow depth of 5 cm or more assumed to be from melting, excluding consistent melt at the end of winter. Mean data for each factor were compared between winters using the statistical analyses outlined in the following section.

### 2.7 Statistical analyses

All statistical analyses and generation of graphs were undertaken using the statistical software R, version 4.1.0 (R Core Team, 2021). Data were nested by site to avoid pseudoreplication and Shapiro‐Wilk normality tests were carried out to verify normality of data.

The impact of stand maturity class was measured for the following dependent variables: lichen biomass, lichen height, lichen cover, understory height, percentage cover of species edible and not edible to reindeer, percentage ground cover of plant litter, percentage cover of bare ground, and canopy cover. When data were normally distributed, the overall impact of stand maturity on these dependent variables was tested using a one‐way ANOVA, followed by a Tukey's post hoc test to provide a more detailed comparison of the impact of each of the four maturity classes. For data that were not normally distributed, this was done using a Kruskal–Wallis test followed by a Dunn's post hoc test. To identify any confounding effects, a scatterplot matrix of correlations for the above variables was constructed.

A factorial ANOVA was then carried out to identify the combined effects of site maturity class, understory height, lichen height, lichen cover, non‐edible species cover and litter cover on lichen biomass.

According to normality of data, either an ANOVA and Tukey's test, or a Kruskal–Wallis and Dunn's test was used to test the impacts of stand maturity on snow depth, number of layers and number of PKI layers. These variables were also tested against the month within which these measurements were taken, these being November, January, and March. For the long‐term dataset of winters starting from 2006 to 2019, a Dunn's test was used to compare the effect of year on days of consistent snow cover, snow depth, mean temperature, and number of days above 0°C.

R packages used in these analyses were FSA, gplots, ggplot2, plotrix and GGally.

## 3 RESULTS

### 3.1 Stand age and understory community composition

Clear‐cut forests (no standing trees) were deemed to be zero years of age. Young stands sampled had a mean age of 7 ± 1 years (standard error), intermediate stands 23 ± 4 years, and old stands 86 ± 13 years. It should be noted that “old” stands here have the monoculture and organized structure of commercial forests, as opposed to the tree diversity, abundance of deadwood, and uneven tree placement found in old‐growth forests.

The average number of plant species per site ranged between 7–10. Mean litter cover, bare ground cover, understory height and percentage cover of species edible to reindeer did not vary significantly according to stand maturity class (Table 1). The percentage cover of non‐edible species decreased significantly between clear‐cut and old sites at 68.7 ± 2.8% and 44.8 ± 4.8%, respectively (Tukey's *p* = .017, Table 1). Canopy cover of trees increased with forest maturity class, peaking in old sites at 70.8 ± 7.0%.

**TABLE 1:** Impacts of variation in forest maturity class in Northern Sweden on characteristics of the understorey vegetation community, notably lichen

	Clear‐cut (C)	Young (Y)	Intermediate (I)	Old (O)	Total replicates	Test	Test Statistic	*p* Value	Post Hoc Test	Significant interactions from Post‐Hoc Test (*p* Value)
Lichen biomass (kg ha^−1^)	416.1 ± 128.1	694.4 ± 201.9	870.4 ± 146.8	828.2 ± 81.4	*n* = 16	ANOVA	**1.97**	.**173**	–	–
Lichen height (cm)	1.6 ± 0.3	1.8 ± 0.3	1.8 ± 0.3	3.7 ± 0.4	*n* = 16	ANOVA	8.0	.003**	Tukey	O‐C (0.006)** O‐Y (0.009)** O‐I (0.011)*
Lichen cover (%)	20.7 ± 4.7	27.3 ± 5.5	29.3 ± 0.9	24.6 ± 3.9	*n* = 16	ANOVA	**0.83**	.**13**	–	–
Arboreal Lichen (kg ha^−1^)	0	0	0	41.5 ± 10.2	*n* = 4	Kruskal–Wallis	14.62	.002**	Dunn	O‐C (0.011)* O‐Y (0.011)* O‐I (0.011)*
Branch Lichen (kg ha^−1^)	0	0	0	9.6 ± 2.7	*n* = 4	Kruskal–Wallis	14.62	.002**	Dunn	O‐C (0.011)* O‐Y (0.011)* O‐I (0.011)*
Understory height (cm)	10.0 ± 1.7	9.6 ± 1.3	9.2 ± 1.1	12 ± 0.9	*n* = 16	ANOVA	**0.94**	.**452**	–	–
Edible species cover (%)	92.0 ± 6.7	112.3 ± 25.7	121.1 ± 19.7	118.7 ±7.3	*n* = 16	Kruskal–Wallis	**4.52**	.**2103**	–	–
Non‐edible species cover (%)	68.7 ± 2.8	55.1 ± 3.7	51.9 ± 6.7	44.8 ± 4.8	*n* = 16	ANOVA	6.28	.024*	Tukey	O‐C (0.017)*
Litter cover (%)	25.0 ± 6.6	9.8 ± 4.2	10.3 ± 4.7	9.9 ± 4.0	*n* = 16	ANOVA	**2.29**	.**503**	–	–
Bare ground cover (%)	13.9 ± 5.1	6.0 ± 2.1	8.2 ± 4.0	1.4 ± 1.0	*n* = 16	Kruskal–Wallis	**5.19**	.**1583**	–	–
Canopy Cover (%)	0 ± 0	4.6 ± 1.3	19.0 ± 7.4	70.8 ± 7.0	*n* = 12	Kruskal–Wallis	12.47	.018*	Dunn	O‐Y (0.015)*

Note

Data was collected in the summer of 2019. Adjust‐*p* values from the post‐hoc tests are shown for sites which differ significantly from one another. All variables had three degrees of freedom. For ANOVAs the test statistic is an *F* value, and for Kruskal‐Wallis tests it is the Chi squared value. Bold values are not statistically significant (*p* > .05). *p < .05; **p < .01.

### 3.2 Lichen abundance

Terrestrial lichen species present were *Cladonia arbuscul*a, *C*. *rangiferina*, *C*. *pyxidata*, *C*. *stellaris*, *Stereocaulon* spp., and *Nephroma arcticum*. Mean lichen biomass and lichen cover did not vary significantly in relation to stand maturity class (ANOVA, *p* > .05, Table 1).

Lichen height was significantly greater in old sites at 3.7 ± 0.4 cm, when compared to all other site maturity classes (Tukey's, *p* < .05 for all, Table 1).

Arboreal lichens were found at all replicates of old sites, and none of the younger forests. The species present were *Hypogymnia physodes* (L.) Nyl. and *Bryoria fuscescens (*Gyeln.) Brodo & D. Hawksw., with a mean weight of 41.5 ± 10.2 Kg ha^−1^, and representing 5% of the total lichen mass present. Lichen mass on wind‐thrown branches was 9.6 ± 2.7 Kg ha^−1^ representing 1.2% of the mean site total (Table 1).

A matrix of correlations showed a significant relationship between a range of factors tested here, notably between lichen biomass and both litter cover and lichen cover, with more extensive details shown in Appendix A. When examining combined effects, lichen biomass varied significantly according to a combination of forest maturity class (*p* < .05), understory vegetation height (*p* < .01, correlation −0.406), and lichen height (*p* < .01, correlation 0.341), but not lichen cover, non‐edible species cover and litter cover (Table 2). This was due to a positive correlation with lichen height (0.341), a negative correlation with understory vegetation height (−0.406), and an increase in biomass from clear‐cut to intermediate/old maturity classes (see Appendix A for more details).

**TABLE 2:** Combined effects of understorey factors on lichen biomass in boreal forests in Northern Sweden, from a factorial ANOVA on data collected during summer 2019

	df	Sum sq	Mean Sq	*F* Value	Pr (>F)
Stand maturity class	3	80,680	26,893	7.741	0.013*
Understory height	1	59,995	59,995	17.269	0.004**
Lichen height	1	56,456	56,456	16.250	0.005**
Lichen cover	1	16,715	16,715	4.811	**0.064**
Non‐edible species cover	1	5,756	5,756	1.657	**0.239**
Litter cover	1	700	700	0.201	**0.667**
Residuals	7	24,319	3,474	–	–

Note

df denotes degrees of freedom. *p < .05; **p < .01.

### 3.3 Snow depth and ice layers

**FIGURE 2:**
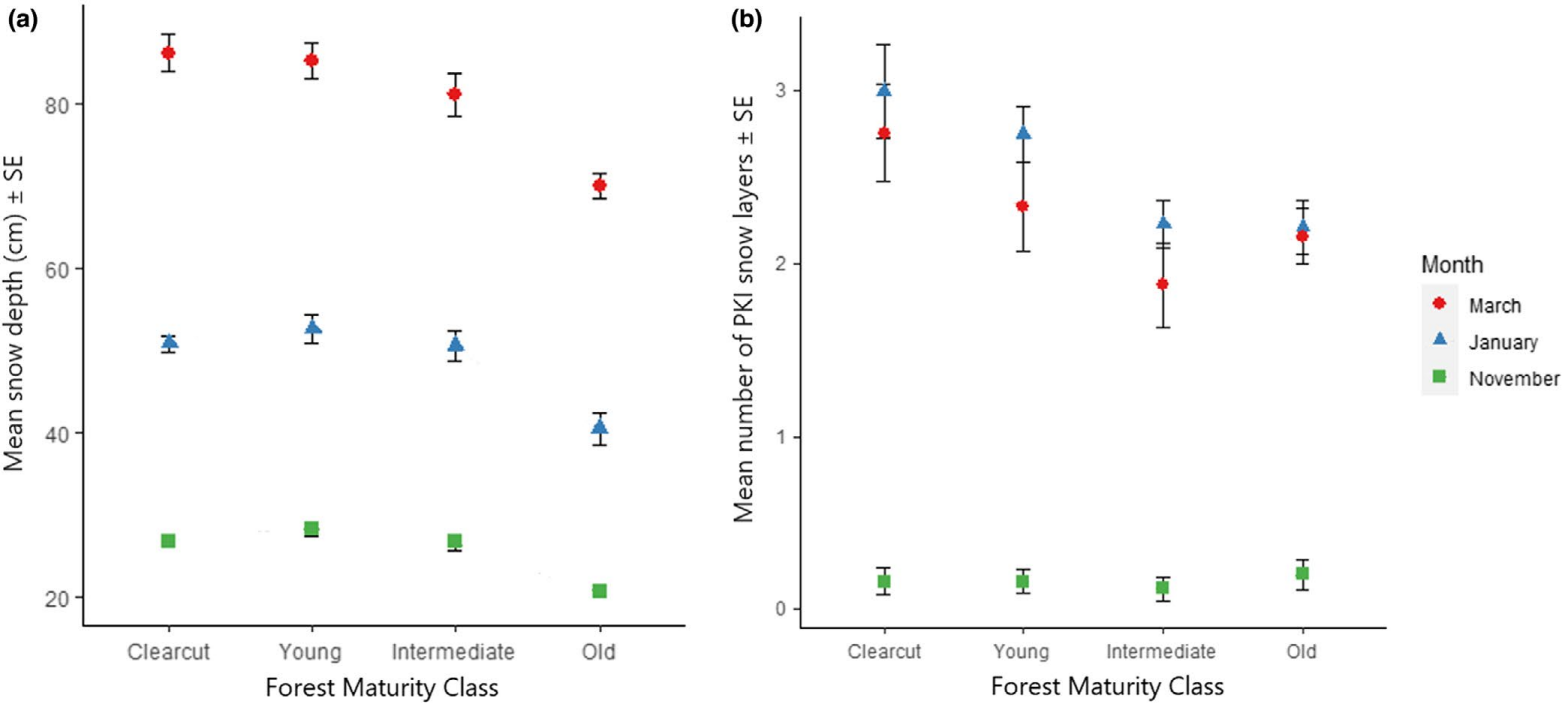
Snow characteristics according to forest maturity class measured throughout the winter of 2019–2020 in boreal forests of Northern Sweden. (a) Shows mean snow depth and (b) mean number of PKI snow layers (impenetrable to reindeer) in the snow column, with bars showing standard errors where large enough to be shown (*n* = 16 per month)

In November and March, old sites had significantly lower snow depth compared to all other sites, at up to 19% shallower (Tukeys Test, *p* < .05 for all, Figure 2a). The other three maturity classes showed little difference in snow depth between each other. The deepest snow in November and January was found in young forests at 28.2 cm and 52.6 cm respectively. In March clear‐cut areas had the deepest snow at 86.2 cm. Snow depth across all sites was shallowest in November at 25.6 ± 0.92 cm, followed by January at 48.7 ± 1.68 cm and March at 80.3 ±1.12 cm (*p* < .01 for one‐way ANOVAs between months).

In November, there were an average of 1.9 distinct layers and in March an average of 7.3 layers of differing hardness in the snow column, which varied little with forest maturity class (ANOVA *p* > .05). In January the number of layers was significantly higher in clear‐cut forests compared to all other maturity classes (mean 6.6 layers, Tukey's Test *p* < .01 for all), varying little between other maturity classes (mean 5.8–6.7 layers).

The number of PKI layers in the snow column did not vary significantly according to stand maturity class (Figure 2b). Overall in November, there were significantly fewer PKI layers at 0.2 ± 0.06 compared to January 2.6 ± 0.12 and March 2.4 ± 0.13 (Dunn's test, all *p* < .001), showing that during November there were notably more accessible snow conditions compared to later in the season. The mean thicknesses of PKI layers did not differ significantly with maturity class, but did increase significantly through time from 0.8 ± 0.3 cm in November to 11.2 ± 0.8 cm in January and 15.8 ± 1.4 cm in March (Dunn's Test, all *p* < .01). In November only “old” forests had PKI layers, which were present in 25% of old stands. Intermediate and clear‐cut stands had PKI layers present at 50% of sites, and young forests at 75% of the stands sampled. January and March saw some PKI layers in all the forest sites sampled.

**FIGURE 3:**
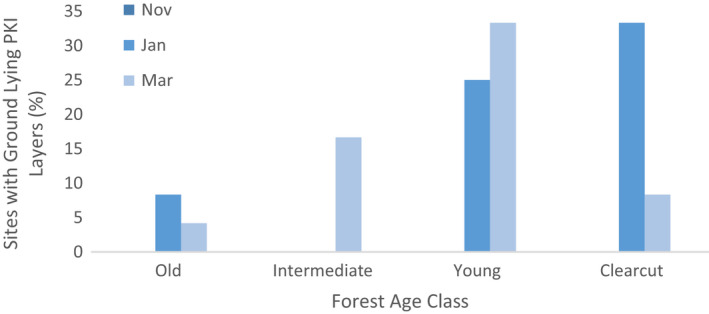
Proportion of forest stands of differing maturity classes with ground lying “båddne” PKI layers, hardened layers of ice deemed difficult to impossible for reindeer to dig through. Measurements were taken in the winter of 2019–2020 in boreal forests of Northern Sweden. No sites had PKI layers in November. (*n* = 24 per maturity class per month)

No sites had *båddne* PKI layers in November. In January they were present in 0% of the intermediate sites, 8.3% of old sites, 25% of young sites, and 33.3% of clear‐cut sites. In March *båddne* ice was present in 4.2% of old sites and 8.3% of clear‐cut sites, a decrease in both though most notably in clear‐cut sites. Intermediate sites had *båddne* PKI layers at 16.7% of sites, and young sites 33.3% (Figure 3).

In January 50% of all clear‐cut, intermediate and old forest sites showed signs of cratering, as did 75% of young sites. In March 50% of old sites also showed signs of cratering, which rose to 75% in young and intermediate sites, but no clear‐cut sites showed recent cratering activity. There were no clear signs of cratering in November at any sites.

### 3.4 Long‐term snow conditions

During the winter of 2019/20, when this research took place, the weather station at Murjek had 205 days of consistent snow cover which did not differ significantly compared to winters starting from 2006 to 2018 (Dunn's Test *p* > .05). Mean snow depth in 2019 differed significantly when compared to only four of the previous 13 years (Dunn's test *p* < .05). Three of these years, these being 2008, 2010, and 2018, differed significantly in snow depth compared to at least 7 of the 13 other winters, indicating they themselves were anomalies in terms of yearly climate condition (Appendix B). The mean temperature at Jokkmokk flygplats weather station in the winter of 2019/20 did not differ significantly from past years at −5.7°C (Dunn's test *p* > .05, Appendix C), although the maximum temperature was 2.7°C cooler at 5.7°C, and the minimum 4.8°C warmer at −20.7°C.

**FIGURE 4:**
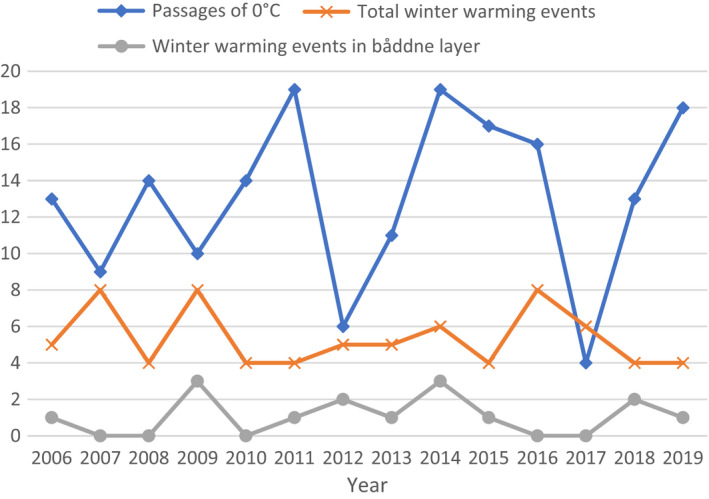
Number of winter warming events from 2006 to 2019, alongside warming events that occurred when 25 cm or less snow was present in the “båddne layer,” which could lead to ground icing. Data are from Murjek D weather station in Northern Sweden. Also shown the number of occasions when temperatures passaged from below to above 0°C each winter

The number of days with temperatures >0°C in the winter of 2019 was 57, which did not differ significantly from previous years (Dunn's test *p* > .05, Figure 4). The temperature crossed from below to above 0°C 18 times in 2019, theoretically providing 18 opportunities for icing to occur (Figure 4). The mean number of layers within the snow at the end of winter was 7.3, although the maximum was 15 found at a clear‐cut stands, showing each passage of 0°C did not necessarily lead to the formation of a new snow layer. All other years ranged between 9 and 19 passages of 0°C, with the exception of 2012 (6 passages) and 2017 (4 passages).

There were 4 winter warming events, with snow melt of 5 cm or greater, during the winter of 2019/20. This was below average for past years, with the winters from 2006 to 2019 showing a mean of 5.4 ± 0.4 warming events per season. When only 25 cm of snow or less was present, only one winter warming event occurred in 2019/2020 which would increase risk for *båddne* icing to occur. The average number of warming events for winters between 2006 and 2009 was 1.1 ± 0.3 (Figure 4). In 2014 one warming event caused snow to completely melt before reforming on 1st November, whilst in 2018 two warming events cause snow to melt completely before snow re‐formed on 1st December. No other winter warming events led to a complete loss of snow.

### 3.5 Forage availability

In November all lichen was accessible for grazing. However, in January 29.2% of lichen was inaccessible for reindeer at clear‐cut sites due to *båddne* PKI layers being present, with 7.3% being inaccessible in March. In young sites, the loss of availability was 25% and 33.3% for January and March, and in intermediate sites, a loss of availability was only seen in March at 16.7%. In old forests, there was an 8.3% and 4.2% loss of access to lichen for reindeer in January and March, respectively. These proportional losses affected the total abundance of lichen available for reindeer to graze throughout winter.

**FIGURE 5:**
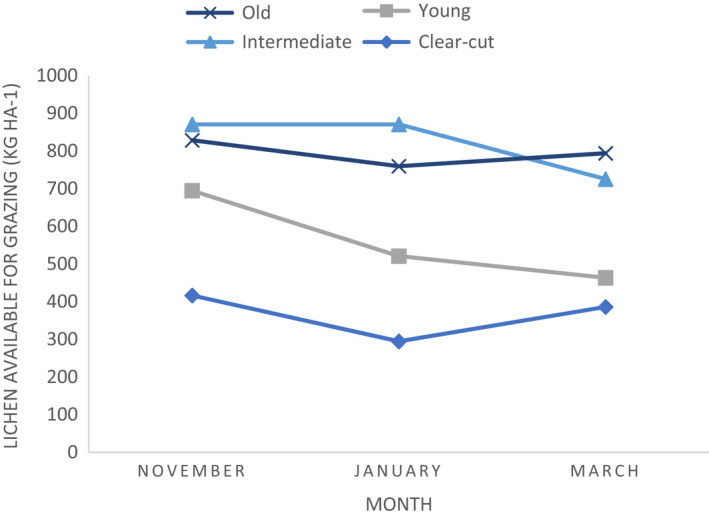
Abundance of lichen present and accessible for reindeer to graze in clear‐cut, young, intermediate, and old forest maturity classes shown throughout the winter of 2019–2020. (*n* = 12)

Combining biomass of lichen present and accessibility of sites due to snow conditions, the total abundance of lichen available for reindeer to graze was as follows: In old sites, the lichen available varied little throughout, ranging between 828 and 759 kg ha^−1^ on average. Intermediate forests had similar available lichen abundance as old forests, of 870 kg ha^−1^, although this decreased in March to 725 kg ha^−1^. Accessible lichen abundance was notably lower in young forests and decreased throughout winter from 694 kg ha^−1^ to 463 kg ha^−1^. Clear‐cut forests had consistently lowest availability of lichen. This decreased between November and January from 416 kg ha^−1^ to 295 kg ha^−1^, although availability rose in March to 386 kg ha^−1^ (Figure 5).

Overall, the abundance of lichen available for grazing at clear‐cut sites is significantly lower when compared to old and intermediate forests. There is 50% less lichen available for grazing in November, 61% in January and 47% in March at the recently logged sites compared to intermediate and old growth forests.

## 4 DISCUSSION

### 4.1 Effects of silviculture disturbance on lichens

Clear‐cutting is known to be detrimental to lichen growth and survival due to soil disturbance caused by machinery during processes such as scarification (Korosuo, 2014; Roturier & Bergsten, 2006; Vanha‐Majamaa et al., 2017). This can reduce lichen cover and biomass by up to 70%, even 15 years after soil preparation has taken place (Roturier et al., 2011), although the severity of these impacts vary (Roturier & Bergsten, 2006). Earlier studies have shown that alternative forestry methods involving less soil disturbance, such as winter harvesting or using gentler forms of scarification compared to the conventional disc trenching, can significantly reduce both the loss of lichen and increase its rate of recovery from disturbance (Coxson & Marsh, 2001; Roturier et al., 2011). Equally, thinning or partial cutting of stands can be beneficial, as it requires use of smaller machines due to limited room to maneuver in the remaining forest, creating less disturbance and encouraging lichen growth on the remaining trees (Korosuo, 2014; Stevenson, 1986; Waterhouse et al., 2011). However, it should be noted that opening the forest canopy can encourage the presence of predators who prey on reindeer, so this option should be explored with care (Fortin et al., 2016; Rasmus et al., 2020).

In this study, the greatest increase in lichen biomass occurred between clear‐cut and young forests. This suggests that the soil disturbance and high prevalence of bare ground immediately after clear‐cutting provides conditions in which lichens have an initial competitive advantage over bryophytes and higher plants, allowing rapid growth and colonization. The role of competition is important to note. Understory vegetation was tallest in old forest sites, and bare ground almost non‐existent, indicating heavier competition for access to space and light. Despite having similar biomass, mean lichen cover decreased between intermediate and old sites, perhaps being outcompeted by competitors such as the mosses *Dicranum scoparium* and *Pleurozium schreberi* (Sulyma & Coxson, 2001).

Whilst open canopies are associated with greater lichen abundance due to increased sunlight (Pharo & Vitt, 2000; Uboni et al., 2019), it has also been seen that opening a mature canopy through thinning does not actually promote the recovery of species important to reindeer grazing (Vitt et al., 2019). Conversely when fires have opened the canopy and suppressed some ground lying vegetation, this has encouraged lichen growth once re‐established (Berg et al., 2008; Cogos et al., 2019; Östlund et al., 1997). Therefore, our results suggest that the disturbance effects of forestry on lichen, and therefore their rate of recovery, are additionally dependent upon the level of competition experienced from surrounding vegetation, with understory vegetation height being a proxy for that competition here. This can be seen in the results that maturity class alone does not significantly impact lichen biomass, but rather it has an impact when combined with the factors of understory height and lichen height.

### 4.2 Variation in snow conditions

A critical factor affecting reindeer grazing in winter is snow (Reinert et al., 2009; Roturier, 2011; Roturier & Roué, 2009). Snow conditions were seen to get more unfavorable in the study area from November to March, with significant increases in snow depth alongside the number and thickness of impenetrable PKI layers within the snow column. This suggests increased energetic costs for reindeer as the season progressed.

In relation to stand maturity class, snow was significantly shallower in old sites and deepest in clear‐cut and young forests, a pattern consistent with previous studies (Horstkotte & Roturier, 2013). Reindeer herders state that the small trees in young forests encourage snow accumulation, whereas in older forests snowfall is trapped in the multilayer canopy, dropping at different times onto the ground (Roturier, 2011). This creates variability in the snow column which allows grazing in a wide variety of snow conditions and thus gives the reindeer more opportunities to adapt to adverse winter weather (Horstkotte & Roturier, 2013; Korosuo et al., 2014).

Whilst the number of ice layers within the snow differed little across maturity classes in November, there were significantly more in clear‐cut sites in January. The absence of trees allows a greater movement of wind, and so greater wind‐hardening of snow can occur compared to older forests sheltered by mature trees (Collins & Smith, 1991; Heggberget et al., 2002). In March the number of ice layers was highly heterogenous within sites so did not differ in a statistically significant manner. This may be as some of the snow held in the canopy of trees had started to drop onto the ground‐lying snowpack, often with force, which may have caused patches to compress into harder layers leading to a greater variation in snow characteristics (Roturier, 2011).

### 4.3 Båddne icing and winter warming events

Herders have specifically highlighted the dangers of icing in the bottom‐most layers of the snow, termed *båddne*. Here no basal PKI ice layers occurred in November, whereas in January 33% of clear‐cut sites and 25% of young sites had this feature. In March, the prevalence of *båddne* icing was 33% in young sites, 17% in intermediate sites, and less than 10% in clear‐cut sites. As some cratering occurred at sites with PKI layers present, only *båddne* PKI layers here were deemed impossible to dig through and therefore used in the later forage availability calculations. However, some areas with multiple PKI layers but no *båddne* ice would also be impenetrable, meaning our estimates of site inaccessibility are likely to be conservative. This icing can create difficulties for the reindeer, who are either forced to remain with insufficient food or are pushed to migrate elsewhere (Stein et al., 2010). If poor snow conditions are extensive, the animals may be restricted by the reindeer herding area boundaries, becoming not only biologically but also politically constrained.

Broadly, long‐term weather data showed greater numbers of winter warming events coincided with greater warming in the *båddne* layer, increasing risk of *båddne* PKI layers forming, yet this was not the case every year. This highlights the importance of examining other factors in the environment which can contribute to *båddne* icing, such as change in forest structure, when studying the relationship between reindeer and snow.

### 4.4 Loss of available forage

Previous studies have broadly explored the effects of forestry on lichen abundance (Akujärvi et al., 2014; Korosuo, 2014; Sandström et al., 2016; Stone et al., 2008; Uboni et al., 2019), and how forest structure alters snow conditions (Roturier, 2011; Roturier & Roué, 2009). However, the real‐life use of forage by reindeer depends upon both forage abundance and its accessibility through snow. When combining these two datasets, a clear pattern emerges. Old forests have a consistent abundance of accessible lichen throughout the season, similar to that in intermediate forests, and remain more accessible throughout winter. Young forests have significantly less lichen available for grazing and this decreases through the season, making them better suited to grazing in early winter, as also seen in Axelsson‐Linkowski et al. (2020). Finally, clear‐cut sites consistently have the lowest available forage for reindeer, partly due to a lower abundance of lichen, and partly due to greater occurrence of basal PKI layers forming a barrier to grazing, deterring reindeer (Collins & Smith, 1991; Horstkotte & Roturier, 2013). Altogether there is up to 61% less forage available in clear‐cut sites than at old sites. These results highlight that for the majority of winter a wide area of older forest stands is necessary. More frequent felling reduces stand age and maturity class, taking away important food resources from reindeer, so perhaps extending the period of time between felling should be considered.

Clear‐cutting through conventional methods remains the dominant form of felling, and “reindeer‐friendly” methods have been argued by some to be uneconomical or to cause opportunity losses when forests are preserved for reindeer (Bostedt et al., 2003). This disconnect between the needs of industry and reindeer herding have led to significant conflicts between these groups, despite mandatory consultations being in place since 1979 (Widmark et al., 2013). However, there are multiple examples of low‐cost solutions that can provide for the needs of both. For example, tree harvesting schedules can be devised in ways which minimize negative impacts on reindeer, creating corridors they can travel through and graze as required whilst allowing tree felling to continue (St John et al., 2016); other beneficial methods such as winter felling or alternative scarification methods have been mentioned earlier (Coxson & Marsh, 2001; Roturier et al., 2011). There is then potential to reconcile some of these differences in ways that benefit reindeer and incur little cost to the forestry industry (Bostedt et al., 2003; Sandström et al., 2006).

An additional point to note relates to arboreal lichens. Amongst reindeer herders, they have often been seen as an emergency food source during periods of severe icing. However, our results reflect those from Korosuo et al. (2014) who stated that the current biomass of arboreal lichen present in forests is so low that it is unlikely to make a significant difference to reindeer survival in poor‐snow conditions. This low biomass could be as a result of multiple poor winters causing reindeer to graze away much of the arboreal lichen, although it could also be impacted by other factors such as the proximity of other stands with arboreal lichen which would be required for spore release to allow lichens to colonize a newly growing stand.

### 4.5 Opportunities and limitations

There are some limitations to the work undertaken in this study. Research was only undertaken during one winter due to unforeseen restrictions in international movement due to the COVID‐19 pandemic. Inter‐annual climatic variations can affect the level of icing through the number of freeze‐thaw and rain‐on‐snow events that occur. Longer term climatic data collated here, including mean winter temperatures, the number of days with temperatures >0°C and mean snow depth, showed that the winter during which fieldwork was undertaken did not differ significantly in climatic conditions from the previous 13 years, aside from years which appear to be anomalous themselves. The number of winter warming events during our field collection is below average for previous years, indicating that at most the results in this study provide a conservative or mild estimate of environmental conditions for a “normal” year. Although climatic data were collected some 32–50 km distance from our field sites, and future studies would benefit from sampling during multiple winters, the broader trends of similarity indicate that the results collected in this study can be regarded as representative of an average winter within this environment.

Another limitation of this study is the volume of data. 16 sites, further divided into four maturity classes of four sites each, formed the basis of the statistical analyses of this research. This allowed a high level of detailed data to be collected both in summer vegetation surveys and winter snow pits, but the trade‐off of such detailed work was inclusion of fewer site replicates.

Plant litter, such as branches dropped after logging, may be a contributing factor to the outcomes discussed above. Mean plant litter presence was notably greater in clear‐cut stands, although its presence was highly variable. In some clear‐cut sites, it covered up to 44% of the ground area and is known to hinder reindeer grazing by causing a barrier between them and terrestrial forage (Eriksson, 1976; Tuorda, 2018). The extent of the barrier effect of litter is unknown but it is likely it would add to accessibility issues, meaning that, in combination with the conservative estimates of which snow conditions are impenetrable to reindeer, in reality even less forage would be available at clear‐cut sites than stated here.

Finally, Rangifer are known to avoid areas that have been recently clear‐cut (Vors et al., 2007). The impacts of this kind of behavioral data on plants through grazing and trampling pressure, as well as on reindeer access to forage, were not included in this study. However, it may form another important component of the ecology of the system.

## 5 CONCLUSION

This study has highlighted the importance of utilizing a broad approach, considering multiple variables, when asking ecological questions. Despite the lower quantity of data produced in this type of research, this approach gives space for a greater level of detail in that data. This allows for improved consideration of combined and cascading effects, whether this be of forage presence and accessibility, or of the interacting relationships within the understory community when faced with disturbance from forestry. Our results have shown that the mechanisms behind the impacts of forestry on reindeer grazing are complex and multifaceted, but that overall clear‐cutting is detrimental to reindeer. Therefore, whilst Sweden has extensive forest cover compared to many other nations, the state of these forests is not necessarily favorable to reindeer (Berg et al., 2008; Kivinen et al., 2012; Korosuo et al., 2014). As levels of silviculture are increasing, this indicates that reindeer and reindeer herding is under growing pressure (Jonsson et al., 2011). To mitigate some of these impacts, the current scale and methods used in forestry in Sweden should be questioned, and alternative low‐cost methods which are beneficial to herding should be explored.

## CONFLICT OF INTEREST

The authors declare no conflicts of interest.

## AUTHOR CONTRIBUTIONS

**Ilona Kater:** Conceptualization (equal); Data curation (lead); Formal analysis (lead); Investigation (lead); Methodology (equal); Project administration (lead); Resources (equal); Validation (equal); Visualization (lead); Writing – original draft (lead); Writing – review & editing (lead). **Robert Baxter:** Conceptualization (equal); Formal analysis (supporting); Funding acquisition (lead); Investigation (supporting); Methodology (equal); Project administration (supporting); Resources (equal); Supervision (lead); Validation (equal); Visualization (supporting); Writing – original draft (supporting); Writing – review & editing (supporting).

## Data Availability

*Permit—*No Permit was required to undertake this work. *Data archive—*Data are available in DataDryad at https://doi.org/10.5061/dryad.1ns1rn8st.

## APPENDIX A

Matrix of correlations for forest stand and understorey vegetation characteristics measured in a boreal forest in northern Sweden during summer 2019. Maturity indicates stand maturity class of ‘old’ (4), ‘intermediate’ (3), ‘young’ (2) and ‘clear‐cut (1). Edible and non‐edible indicate percentage cover of species relative to their palatability to reindeer. Litter and bare indicate ground cover and canopy is measured in percentage cover. **p* < .05; ***p* < .01.

## APPENDIX B

Mean daily snow depth measured between 1st October and 20th April for years starting 2006 to 2019, taken at Murjek D weather station (SMHI, 2021b). Starting date of each year's measurement shown on x‐axis.

## APPENDIX C

Mean daily temperature measured between 1st October and 20th April for years starting 2006 to 2019, taken at Jokkmokk flygplats weather station (SMHI, 2021a). Starting date of each year's measurement shown on x‐axis.
